# Editorial: Preprints, citations and *Nucleic Acids Research*

**DOI:** 10.1093/nar/gky1229

**Published:** 2018-12-18

**Authors:** Barry L Stoddard, Keith R Fox

**Affiliations:** 1 Division of Basic Sciences, Fred Hutchinson Cancer Research Center, 1100 Fairview Ave. N. A3-025, Seattle, WA 98109, USA; 2 Biological Sciences, Life Sciences Building 85, University of Southampton, Southampton SO17 1BJ, UK

The Editors of *Nucleic Acids Research* are committed to the accurate and efficient dissemination of scientific research to the widest possible audience. To achieve these goals, we employ an editorship that is entirely comprised of active researchers, strive to conduct fair and rigorous peer review in a timely manner and operate as a completely open-access journal.

Over the past 14 years, various online venues for the presentation of preprints in the biological sciences have been established, culminating in late 2013 with the creation of *bioRxiv* (1,2). Since then, the total number of newly posted preprints at *bioRxiv* and similar platforms has risen dramatically (2), with no indication of a slow-down in the immediate future,

The benefits of publically available preprints are obvious. New approaches and results are rapidly disseminated to the research community, thereby accelerating the flow of information and the forward momentum of biological research. The ability of a preprint server to facilitate real-time feedback and dialogue, often within days or even hours of posting, provides tangible benefits to authors and readers alike. Investigators can establish a clearly defined moment of discovery that coincides with their decision to release details of a study and its results. This is of particular importance for young investigators in fast-moving, competitive fields, where name recognition can play a significant role in the pace and outcome of peer review. Preprints are also extremely useful for presenting essential unpublished information in grant proposals and progress reports to funding agencies.

*Nucleic Acids Research* (NAR) has always supported the use of preprint servers as part of the overall process of disseminating and publishing results. We do not place any restrictions on the posting of manuscripts on such platforms before submission to our journal, and do not treat such postings as prior publications. We do not prohibit or discourage public discussion of preprints with the research community or with the press, prior to publication of the same study within our own pages. In cases where potential reviewers indicate that they have previously seen a submission via a preprint server and ask if they should be disqualified from review, we only ask if they are still able to conduct a fair, rigorous and timely evaluation of the study as submitted to NAR.

Until now, NAR has prohibited the formal citation of preprints within the reference list of published articles. Authors have been encouraged, however, to describe and discuss posted preprints (including links to their DOIs) in the main text of the paper, in the same manner that authors are allowed to mention personal communications and/or describe unpublished results and other informal online content that is pertinent to the subject of their study. The reason for this editorial policy has been our belief that formal citations should be reserved for publications that have been subjected to editor-supervised peer review. We also allow citation of textbooks, chapters, databases and on-line web servers that research communities broadly agree provide authoritative information and data within a relevant subject area.

However, other journals in the biological sciences are now beginning to allow citation of preprints within the main reference list. Given that the academic publishing community appears to be moving towards a policy of allowing formal citation of preprints, the Executive Editors of NAR have decided to follow this shift in policy, and to allow their citation. Such references will be required to be presented with the disclaimer ‘PREPRINT: NOT PEER REVIEWED’ as an obvious part of the citation. This change in our policy will be accompanied by very clear instructions to both authors and reviewers that over-reliance on unreviewed material can result in negative evaluation of the manuscript under consideration at NAR.

Some proponents of preprints may argue against this policy, pointing out that the process of peer review does not absolutely guarantee a manuscript’s accuracy. They argue that, since perfection isn’t possible, there is no need to enforce an arbitrary line in the sand regarding citable scientific findings. Some proponents of preprint citations describe prominent examples of failed peer review, contrasted with examples of obviously successful preprint examinations by the public.

The editors at NAR disagree with these arguments. The process of formal, editorially supervised peer review, while clearly not infallible, obviously elevates the overall quality of the published research literature. Missing controls, over-interpreted results and incorrect or flawed studies are often flagged, and the studies are corrected or abandoned. A properly conducted peer review, involving multiple experts who have been specifically commissioned to examine a submission in depth (and who have been provided with specific editorial queries) is more systematic and rigorous than a less formal system of public commentary, which generally does not enforce a requirement for authors to address readers’ concerns and queries.

The formal citation of any research is a serious responsibility for any author and has the effect of validating that article’s contents and conclusions. More than ever, the publishing community is expected to be extremely vigilant with regard to weaknesses in submitted manuscripts (3,4). As a result, we (like many other journals) have created a growing list of expectations and requirements for certification by authors (and corresponding inspection by reviewers). This includes validation of the collection of experimental replicates and the proper use of statistical methods, outlining and enforcing community standards of data collection, analyses and deposition, and careful analyses of submitted images.

Formal citation of a preprint elevates that work to a position that appears equivalent to a peer-reviewed published article. This could place scientific journals and editors at cross-purposes with the type of efforts to improve the reproducibility and quality of the published literature that are described above. If we are expected to reduce the amount of irreproducible and flawed science in the literature, then allowing the formal citation of online postings will require that their quality should also be carefully assessed during the peer review process.

Finally, we offer (and/or reiterate) several recommendations to preprint servers and the research community for the future use and curation of preprints. All preprints should be date- and time-stamped and provided with corresponding version numbers, that would all be included in any formal citations. All versions of any preprint should be maintained in perpetuity at the relevant server and URL, along with a clear description of any ‘subsequent versions’ that indicates the exact nature of any changes to the content and conclusions relative to its predecessor. Any and all online versions of any preprint that eventually lead to a peer-reviewed publication should provide an automated link directly to the actual peer-reviewed publication. Finally, any preprint that has not found its way into the peer-reviewed published literature after a certain period of time (we recommend 18 months) should be amended with an explanation of the circumstances that have precluded its review and publication. Given that the overwhelming majority of preprints in the biological sciences do eventually find their way into the peer-reviewed published literature, this should not impose a significant or frequent burden on researchers and authors.

The current mechanism of posting preprints online, and now allowing their formal citation, is driven by the well-intended desire to accelerate and democratize the dissemination of scientific results—a goal that is shared by *Nucleic Acids Research*. We employ an editorial staff of active researchers, achieve extremely fast and efficient peer review, and fully support publication of research studies that refute or modify conclusions that were previously presented within our pages. We believe that there is no obvious reason why a high quality-journal cannot employ all the forms of quality-control that accompany rigorous peer review, without sacrificing expeditious and fully open access publication of our content to the community of scientists who depend on both factors in their daily work lives.
